# Global left ventricular function quantification with CMR 4D Flow

**DOI:** 10.1186/1532-429X-18-S1-P308

**Published:** 2016-01-27

**Authors:** Raluca G Saru, Kevin Wanambiro, Albert Hsiao, Sara Boccalini, Adriaan Coenen, Ricardo Budde, Piotr Wielopolski, Shreyas Vasanawala, Jolien Roos-Hesselink, Koen Nieman

**Affiliations:** 1 grid.5645.2000000040459992XRadiology and Cardiology, Erasmus MC, Rotterdam, Netherlands; 2 grid.266100.30000000121074242University of california, San Diego, CA USA; 3 grid.168010.e0000000419368956Stanford University, Palo Alto, CA USA

## Background

4D MR flow is a rapidly evolving technique, offering both anatomical and functional information in just a single acquisition and if successful, in the future, the 4D flow sequence should replace the 2D MR sequences. The purpose of this study is to use the anatomical information from the 4D flow sequence to assess the global left ventricular function and compare these results with the ones obtained from standard cine acquisitions.

## Methods

Between September 2014 and February 2015, we prospectively included 22 consecutive adult patients (4 females, mean age 39 years) planned for CMR with a clinical indication for contrast administration. The 4D flow raw data sets were uploaded to a dedicated web-based software application (Arterys Inc., San Francisco, CA, USA).

Images were reconstructed in 20 cardiac temporal phases separately with a compressed sensing algorithm. The end-diastolic, end-systolic and stroke volumes and ejection fraction were measured by CMR 4D flow and compared against cine CMR measurements.

## Results

The mean end-diastolic, end-systolic stroke volumes and ejection fraction were 163( ± 30) ml, 77( ± 34) ml, 85 ( ± 17) ml and 54 ( ± 11)% respectively for CMR 4D flow and 182 ( ± 50) ml, 89 ( ± 42) ml, 93 ( ± 18) ml, 53 ( ± 10)% respectively for cine CMR. The Pearson's correlations between CMR 4D flow and CMR were 0.93, 0.96, 0.77 and 0.93 for end-diastolic, end-systolic, stroke volumes and ejection fraction respectively.

When applying a threshold of 50% for ejection fraction, 21 out of 22 patients were correctly classified.

## Conclusions

In this study we showed that global left ventricular function can be quantified accurately using CMR 4D flow imaging analysed using a cloud based software.

